# Machine learning models for screening carotid atherosclerosis in asymptomatic adults

**DOI:** 10.1038/s41598-021-01456-3

**Published:** 2021-11-15

**Authors:** Jian Yu, Yan Zhou, Qiong Yang, Xiaoling Liu, Lili Huang, Ping Yu, Shuyuan Chu

**Affiliations:** 1 Department of Endocrinology, Affiliated Hospital of Guilin Medical University, Guilin, Guangxi China; 2 Department of Respiratory and Critical Care Medicine, Affiliated Hospital of Guilin Medical University, Guilin, Guangxi China; 3 Laboratory of Respiratory Disease, Affiliated Hospital of Guilin Medical University, Guilin, 541001 Guangxi China

**Keywords:** Machine learning, Cardiology

## Abstract

Carotid atherosclerosis (CAS) is a risk factor for cardiovascular and cerebrovascular events, but duplex ultrasonography isn’t recommended in routine screening for asymptomatic populations according to medical guidelines. We aim to develop machine learning models to screen CAS in asymptomatic adults. A total of 2732 asymptomatic subjects for routine physical examination in our hospital were included in the study. We developed machine learning models to classify subjects with or without CAS using decision tree, random forest (RF), extreme gradient boosting (XGBoost), support vector machine (SVM) and multilayer perceptron (MLP) with 17 candidate features. The performance of models was assessed on the testing dataset. The model using MLP achieved the highest accuracy (0.748), positive predictive value (0.743), F1 score (0.742), area under receiver operating characteristic curve (AUC) (0.766) and Kappa score (0.445) among all classifiers. It’s followed by models using XGBoost and SVM. In conclusion, the model using MLP is the best one to screen CAS in asymptomatic adults based on the results from routine physical examination, followed by using XGBoost and SVM. Those models may provide an effective and applicable method for physician and primary care doctors to screen asymptomatic CAS without risk factors in general population, and improve risk predictions and preventions of cardiovascular and cerebrovascular events in asymptomatic adults.

## Introduction

Carotid atherosclerosis (CAS) is a chronic disease with pathological thickening in common or internal carotid intima^1^. It significantly increases the risk of ischemic stroke, coronary event and airflow limitation^2–4^. The global prevalence of CAS among people aged 30–90 years is estimated to be 27.6% in 2020, namely more than one billion people suffered this disease^5^. In China, the prevalence of carotid plaques is about 31% among general population, and is 39% at the age of 60–69 years^6^. CAS is usually asymptomatic unless the patients suffered symptomatic ischemic stroke, transient ischemic attack, or amaurosis fugax^1^. Thus, CAS put a great health burden worldwide. If CAS in asymptomatic adults can be detected, it could improve risk predictions and preventions of cardiovascular and cerebrovascular events, particularly cardiovascular death, myocardial infarction and ischemic stroke^7^.

Carotid duplex ultrasonography is a noninvasive, safe and easily applicable diagnostic tool for detecting CAS, and has been widely used in CAS diagnosis^8^. However, carotid duplex ultrasonography is not recommended in routine screening for asymptomatic subjects who have no clinical manifestations or risk factors of atherosclerosis^9–11^. Thus, we aim to develop classification model to screen the asymptomatic CAS based on data of routine physical examination from general population, which could help prevent cardiovascular and cerebrovascular events in asymptomatic population.

In recent years, machine learning has been widely used in medical study, and holds the promise to automatically diagnose heterogeneous diseases with high accuracy^12^. It’s also successfully used in studies on CAS and cardiovascular disease^13–15^. In this study, we will develop models using decision tree, random forest (RF), extreme gradient boosting (XGBoost), support vector machine (SVM) and multilayer perceptron (MLP) based on the data from general population without symptoms of CAS. We’ll assess the performance of those models and select good one. Those models will help to screen CAS in asymptomatic adults.

## Results

### Subjects characteristics

A total of 2732 subjects were included in the study, among which 942 (34.5%) subjects were diagnosed as CAS. Compared with Non-CAS group, CAS group was in older age (CAS group vs Non-CAS group: 56.3 ± 7.4 vs 49.4 ± 6.8 yrs), and had higher blood pressure (systolic blood pressure (SP): 132 ± 20 vs 123 ± 18 mmHg; diastolic blood pressure (DP): 80 ± 12 vs 76 ± 12 mmHg), higher blood uric acid (UA) level (376.5 ± 96.9 vs 352.7 ± 93.8), higher homocysteine (HCY) level (13.22 ± 5.86 vs 11.70 ± 5.31 μmol/L), and worse renal function (blood urea nitrogen (BUN): 5.1 ± 1.5 vs 4.7 ± 1.2 mmol/L; serum creatinine (Scr): 81.64 ± 21.80 vs 76.81 ± 16.10 μmol/L), (Table 1). Moreover, CAS group had a higher proportion of males and the subjects with nonalcoholic fatty liver disease (NAFLD), compared with Non-CAS group (Table 1).

**Table 1:** Subjects characteristics in CAS group and non-CAS group.

Variables	CAS group (n = 942)	Non-CAS group (n = 1790)	*P* values
Gender (male)	686 (72.8%)	1058 (59.1%)	< 0.001
Age (years)	56.3 ± 7.4	49.4 ± 6.8	< 0.001
BMI (kg/m^2^)	25.1 ± 3.0	24.7 ± 3.1	0.003
SP (mmHg)	132 ± 20	123 ± 18	< 0.001
DP (mmHg)	80 ± 12	76 ± 12	< 0.001
AST (U/L)	20.87 ± 7.86	20.22 ± 7.27	0.031
ALT (U/L)	22.94 ± 12.53	22.35 ± 13.38	0.269
BUN (mmol/L)	5.1 ± 1.5	4.7 ± 1.2	< 0.001
Scr (μmol/L)	81.64 ± 21.80	76.81 ± 16.10	< 0.001
TG (mmol/L)	1.93 ± 1.87	1.72 ± 1.48	0.003
TC (mmol/L)	4.89 ± 0.87	4.74 ± 0.83	< 0.001
LDL-C (mmol/L)	3.29 ± 0.81	3.14 ± 0.81	< 0.001
HDL-C (mmol/L)	1.24 ± 0.35	1.29 ± 0.33	< 0.001
UA (μmol/L)	376.5 ± 96.9	352.7 ± 93.8	< 0.001
HCY (μmol/L)	13.22 ± 5.86	11.70 ± 5.31	< 0.001
FPG (mmol/L)	5.88 ± 1.68	5.47 ± 1.21	< 0.001
NAFID (Yes)	285 (30.3%)	417 (23.3%)	< 0.001

CAS, carotid atherosclerosis; BMI, body mass index; SP, systolic blood pressure; DP, diastolic blood pressure; AST, serum aspartate aminotransferase; ALT, serum alanine aminotransferase; BUN, blood urea nitrogen; Scr, serum creatinine; TG, triglyceride; TC, total cholesterol; LDL-C, low-density lipoprotein cholesterol; HDL-C, high-density lipoprotein cholesterol; UA, blood uric acid; HCY, homocysteine; FPG, fasting plasma glucose; NAFLD, nonalcoholic fatty liver disease.

### Model performance

**Figure 1:**
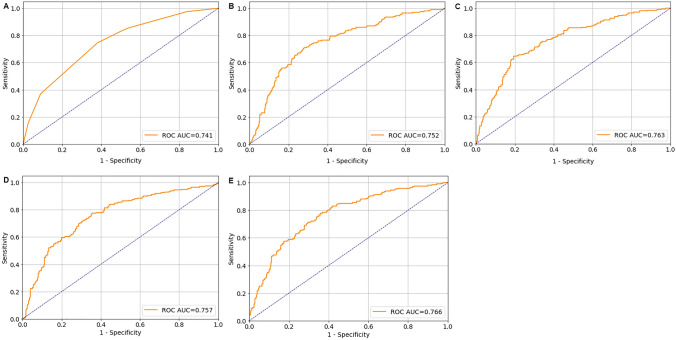
Receiver operator characteristic curves. (**A**) Decision tree. (**B**) Random forest. (**C**) Extreme gradient boosting. (**D**) Support vector machine. (**E**) Multilayer perceptron.

As Table 2 illustrated, the model using MLP showed the best performance among all classifiers with the highest accuracy (0.748), positive predictive value (PPV) (0.743), F1 score (0.742), area under receiver operating characteristic curve (AUC) (0.766) and Kappa score (0.445). The second-best performance was from models using XGBoost and SVM. They were showed very similar performance in testing data. The model using XGBoost showed a bit higher of F1 score (XGBoost vs SVM = 0.735 vs 0.733), AUC (0.763 vs 0.757), and Kappa score (0.429 vs 0.413) than using SVM. The model using RF showed worse performance than using SVM and XGBoost. And the worst performance was from model using decision tree. The receiver operating characteristic curves (ROCs) of all models were showed in Fig. 1

.

**Table 2:** Model performance in testing data according to ranking.

Model	Accuracy	PPV	F1 score	Kappa score	AUC (95% CI)
MLP	0.748	0.743	0.742	0.445	0.766 (0.754–0.769)
XGBoost	0.741	0.736	0.735	0.429	0.763 (0.724–0.764)
SVM	0.744	0.739	0.733	0.413	0.757 (0.718–0.757)
Random forest	0.730	0.724	0.722	0.401	0.752 (0.734–0.766)
Decision tree	0.726	0.723	0.706	0.354	0.741 (0.699–0.749)

PPV, positive predictive value; AUC, area under curve; CI, confidence interval; MLP, multilayer perceptron; SVM, support vector machine; XGBoost, extreme gradient boosting.

### Important features from the models

**Figure 2:**
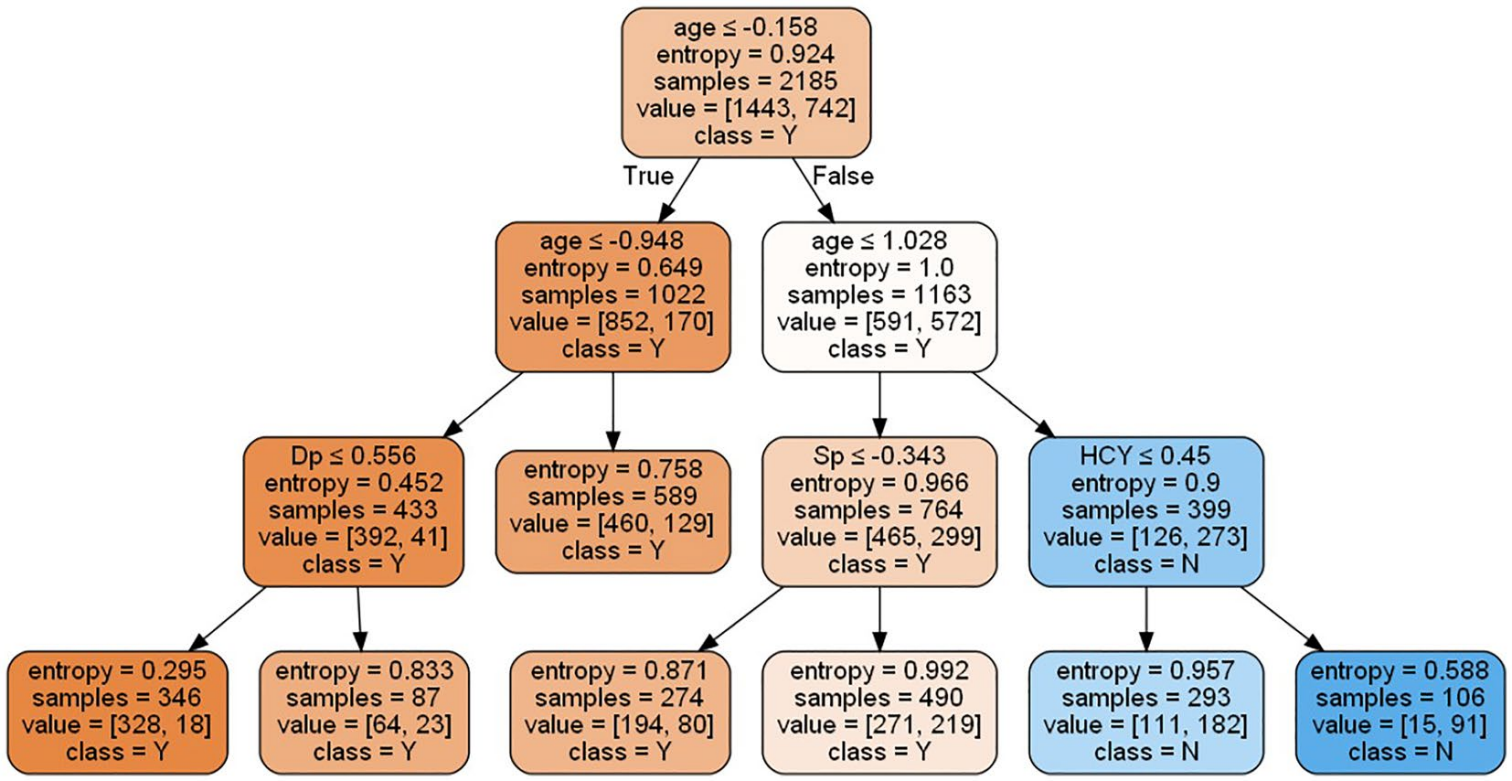
Decision tree. DP, diastolic blood pressure; SP, systolic blood pressure; HCY, homocysteine; Y = Yes; N = No.

**Figure 3:**
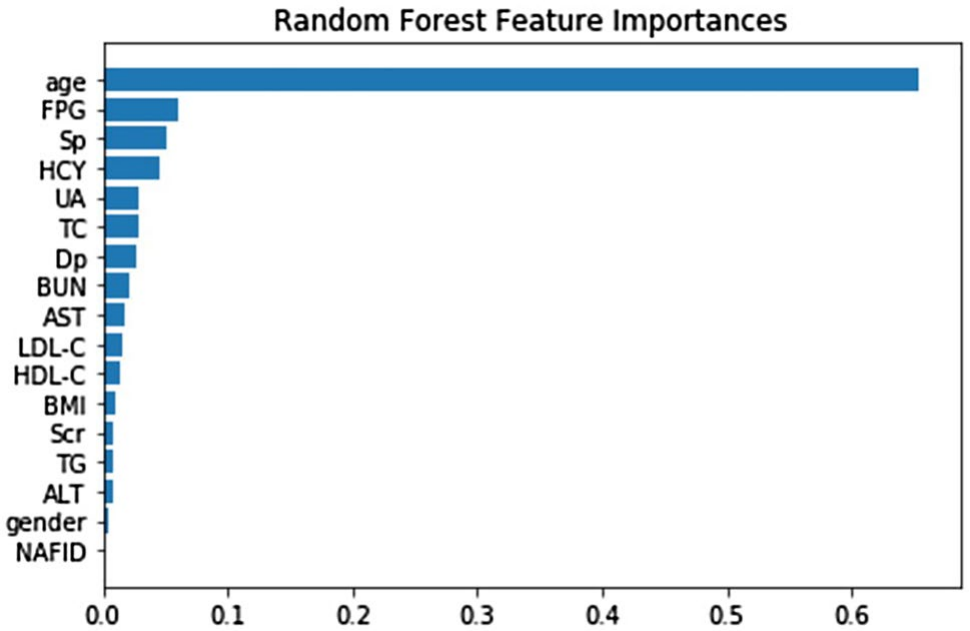
Features importance in random forest model. FPG, fasting plasma glucose; SP, systolic blood pressure; HCY, homocysteine; UA, blood uric acid; TC, total cholesterol; DP, diastolic blood pressure; BUN, blood urea nitrogen; AST, serum aspartate aminotransferase; LDL-C, low-density lipoprotein cholesterol; HDL-C, high-density lipoprotein cholesterol; BMI, body mass index; Scr, serum creatinine; TG, triglyceride; ALT, serum alanine aminotransferase; NAFLD, nonalcoholic fatty liver disease.

**Figure 4:**
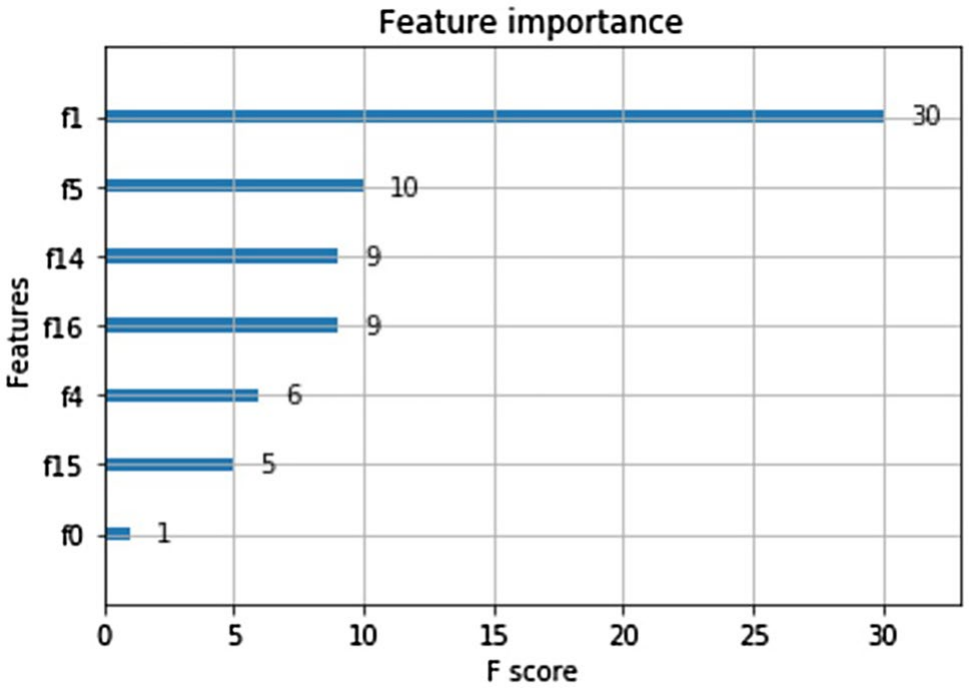
Features importance in XGBoost model. f1: age; f5: Dp; f14: HDL-C; f16: HCY; f4: Sp; f15: FPG; f0: gender.

In this study, classifiers using decision tree, RF, XGBoost and SVM could show the important features in the model. In classifier using decision tree, age was the most important feature, followed with Dp, Sp and HCY (Fig. 2). Since the maxed depth was three in the decision tree from grid-search and tenfold cross-validation, those four features were selected as most important from the model. In classifier using RF, all features could be ranked based on the importance in the model. As showed in Fig. 3, the most important feature was age, followed by fasting plasma glucose (FPG), Sp, HCY, UA, total cholesterol (TC), Dp, BUN, serum aspartate aminotransferase (AST), low-density lipoprotein cholesterol (LDL-C), high-density lipoprotein cholesterol (HDL-C) and others. In classifier using XGBoost, the features selected from model were age, Dp, HDL-C, HCY, Sp, FPG and gender (Fig. 4). In classifier using SVM, the features could be selected using the support vector machine recursive feature elimination (SVM-RFE) algorithm^16^, which could optimize the performance of the classifier. The selected features were age, gender, Sp, Dp, TC, HDL-C, AST and ALT, which were in the same importance without further ranked in the SVM-RFE.

## Discussion

In this study, we developed models using decision tree, RF, XGBoot, SVM and MLP to classify subjects with CAS from asymptomatic adults based on data of routine physical examination. All models were assessed by accuracy, PPV, F1 score and AUC. The best performance was from model using MLP, followed by XGBoost and SVM.

Although carotid duplex ultrasonography is used in CAS diagnosis, there’s no evidence to support the routine ultrasonography screening among general subjects without symptoms or risk factors^9–11^. However, CAS is usually asymptomatic until it leads to serious outcomes, such as cardiovascular and cerebrovascular accident^1^. Considering the high prevalence of CAS^5,6^, it’s necessary to propose an effective, noninvasive and convenient method for screening asymptomatic subjects. Machine learning model from our study is such a method, which could improve risk predictions and preventions of cardiovascular and cerebrovascular events in asymptomatic adults. That may have important clinical and public health implications.

In our study, we developed models using decision tree, RF, XGBoost, SVM and MLP. MLP is artificial neural network, which usually showed good performance (i.e. high accuracy, PPV and AUC) among machine learning models. However, MLP can’t show the important features in the model, or can’t be explained^17–19^. In contrast, decision tree could show the features with visualization and can be explained. In our study, criterion = entropy was selected with grid-search in the decision tree, which means C4.5 tree was developed. RF and XGBoost integrate many decision trees to promote the efficiency and accuracy of a signal tree^18^. Moreover, SVM is also a strong classifier in medical research, which could show important features in the model^20^. Thus, in addition to MLP, we developed models using decision tree, RF, XGBoost and SVM.

For all models in our study, the model using MLP showed the best performance with highest accuracy, PPV, F1 score, AUC and Kappa score. MLP is a neural network with one or more layer of neurons linked together through weighted synapses, in which learning takes place through the backpropagation of the network output error and updating the weights^21^. In our study, the single-hidden layer MLP (hidden_layer_sizes = (100, )) showed the best performance. Although MLP could include multiple-hidden layer, a model with single-hidden layer with enough nodes and right set of weights can learn any function and get the best results, which moreover could run faster than that with multiple-hidden layer^22^. Followed MLP, the model using SVM showed good performance. SVM is an effective approach for classification by using linear functions or special nonlinear functions, namely kernels, to transform the input space into a multidimensional space^23^. Thus, the model using SVM is a good classifier^18^, which was confirmed in our study.

In addition, models using XGBoost and RF performed better than decision tree in our study, since both XGBoost and RF integrate decision trees to promote performance of signal tree model^15,18^. Moreover, the performance of model with XGBoost was similar with that using SVM, and was better than that based on RF in our study. For the principle of algorithm, XGBoost is a library based on the gradient increase framework^24–26^. In contrast, RF is a combination of multiple tree predictions, in which each tree depends on the values of a randomly sampled independent vector^27^. And all trees have the same distribution in the forest^27^. Thus, the model using XGBoost could promote performance more efficient than the one using RF, and then perform better than RF model.

Among all models in our study, models using decision tree, RF, XGBoost and SVM could show important features. Our results showed that age, Sp, Dp, HCY level and HDL-C level were most important in all those four models, followed by gender, TC level and FPG level. Our findings were in consistent with previous studies, in which older age, gender, high Sp, hypertension, high TC level and high FPG levle were independently related to the risk of CAS^5,28–30^. High HCY level was also associated with the progression of CAS^31^. And high HDL-C level was a protective factor for CAS reported in a study with Chinese population^30^. Thus, models using decision tree, RF, XGBoost and SVM in our study suggested that age, Sp, Dp, HCY level, HDL-C level, gender, TC level and FPG level should be important in screening CAS in general and asymptomatic adults.

We acknowledged the limitation in our study that smoking history was not included in candidate features for developing models. It’s widely accepted that smoking is a risk factor for CAS^5,28–30^. However, no record of smoking history in our study. That may reduce the performance of our models, in which the AUC, accuracy, PPV and F1 score were less than 0.8, even in the best model using MLP. Thus, if smoking history was included in models, the performance should be improved.

In conclusion, it could create classification models using machine learning based on the results of routine physical examination. Those classifiers could screen CAS in asymptomatic adults without redundant examination. The model using MLP is the best one, followed by using XGBoost and SVM. Those models may provide an effective and applicable method for physician and primary care doctors to screen asymptomatic CAS without risk factors in general population, which could improve risk predictions and preventions of cardiovascular and cerebrovascular events in asymptomatic adults.

## Subjects and methods

### Study population

The subjects were recruited into this study from general people who took routine physical examination in the Center of Health Examination, Affiliated Hospital of Guilin Medical University, from July to October in 2017. All laboratory testing and quality control were carried out by the laboratory analysis center of our hospital. The study protocol was approved by the Research Ethics Committee of the Affiliated Hospital of Guilin Medical University, and conformed to the declaration of Helsinki. Written informed consent was obtained from each subject.

The inclusion criteria for subjects in the study were as following: (1) male or female; (2) age ≥ 20 years; (3) subjects underwent carotid duplex ultrasonography; (4) subjects received blood testing on liver function, renal function, triglyceride (TG), TC, lipoprotein, HCY and FPG.

The exclusion criteria for subjects were: (1) had clinical manifestations of CAS including ipsilateral amaurosis fugax, retinal infarction, symptomatic ischemic stroke, or transient ischemic attack; (2) had a history of coronary atherosclerosis or coronary heart disease; (3) had autoimmune disorders; (4) had psychiatric disorders; or (5) had malignant tumor.

### Diagnostic criteria

The CAS was determined if carotid intima-media thickness (CIMT) ≥ 1 mm with or without atherosclerotic plaque^32^. The CIMT was automatically measured on the far wall of the left common carotid artery 10 mm proximal to the carotid bifurcation at end-diastole^32,33^ using color Doppler ultrasound with a 7.5-MHZ probe (DC-6 Expert, Mindray, Shenzhen, China) by exporters with at least 5 years’ experience. NAFLD was diagnosed when there was evidence of hepatic steatosis by color Doppler ultrasound with a 3.5-MHZ probe (DC-6 Expert, Mindray, Shenzhen, China) and there was no history of significant alcohol consumption, use of steatogenic medication, viral hepatitis, or hereditary disorders^34^.

### Candidate features to classify subjects

The candidate features were collected from the electronic medical record. They were age, gender, NAFLD (Yes/No), BMI (BMI = weight/height^2^), SP, DP, UA, BUN, Scr, AST, serum alanine aminotransferase (ALT), TG, TC, LDL-C, HDL-C, FPG, and HCY.

### Machine learning classifiers

In each group of subjects, 80% were randomly selected (training sample), who were used to develop the model. The remaining 20% (testing sample) served to test the model. The training data were standardized using z-score transformation, and the testing data were also transformed using the same parameters as those from the training data.

The models were developed using Python3.7.6 programming language (http://www.python.org), scikit-learn 22.2 library (https://scikit-learn.org/stable/). We developed models to classify subjects with CAS or without using decision tree, RF, XGBoost, SVM and MLP. The grid-search and tenfold cross-validation were used to estimate hyper parameters with training dataset. When several parameter combinations were optimal and the choice affected the efficiency of the model, we choose parameter combination which led to the highest efficiency. The hyper parameters of model using decision tree were max_depth = 3, max_leaf_nodes = 7 and criterion = entropy; RF were n_estimators = 10, max_depth = 5, min_samples_split = 76, min_sample_leaf = 35, max_features = 7; XGBoost were max_depth = 3; n_estimators = 100; learning rate = 0.1; SVM were kernal = rbf; C = 1.0; and MLP were hidden_layer_sizes = (100), activation = logistic, solver = adam, alpha = 0.1, max_iter = 100 (Supplement Table 1).

The performance of classifiers was assessed on the testing dataset, which was not used during the training step. The performance of models was assessed using accuracy, PPV, F1 score, AUC and Kappa score.

### Statistical analysis

The continuous variables between case and control groups were analyzed with independent-samples t-test, and the categorical data were compared with Chi-square test. P-values < 0.05 were considered to be statistically significant. Data were analyzed using SAS 9.4 (SAS Institute Inc., Cary, NC, USA).

## Supplementary Information

Supplementary Information.
