# Supplementary material for: Machine learning models for screening carotid atherosclerosis in asymptomatic adults
Source: Sci Rep. 2021 Nov 15;11:22236. doi: 10.1038/s41598-021-01456-3 (PMC8593081; doi:10.1038/s41598-021-01456-3)
Supplement: Supplementary file 1 — Supplementary Information. [file 41598_2021_1456_MOESM1_ESM.docx]

Supplement Table 1. The hyper parameters of decision tree, random forest, XGBoost, SVM and MLP.

| Model | Optimum Value |
| --- | --- |
| Decision tree | criterion = entropy; max_depth = 3; max_leaf_nodes = 7 |
| Random forest | n_estimators = 10; max_depth = 5; min_samples_split = 76; min_sample_leaf = 35; max_features = 7 |
| XGBoost | max_depth = 3; n_estimators = 100; learning rate = 0.1 |
| SVM | kernal = rbf; C = 1.0 |
| MLP | hidden_layer_sizes = (100,); solver = ’adam’; alpha = 0.1; max_iter = 100 |

Abbreviations: XGBoost, extreme gradient boosting; SVM, support vector machine; MLP, multilayer perceptron
